# Phase Separation of Intrinsically Disordered Nucleolar Proteins Relate to Localization and Function

**DOI:** 10.3390/ijms222313095

**Published:** 2021-12-03

**Authors:** Francisco Guillen-Chable, Andrea Bayona, Luis Carlos Rodríguez-Zapata, Enrique Castano

**Affiliations:** 1 Biochemistry and Molecular Plant Biology Department, Centro de Investigación Científica de Yucatán A.C., Calle 43 No. 130, Colonia Chuburná de Hidalgo, Mérida C.P. 97200, Yucatán, Mexico; francisco.guillen@cicy.mx (F.G.-C.); andrea.bayona@estudiantes.cicy.mx (A.B.); 2 Biotechnology Department, Centro de Investigación Científica de Yucatán A.C., Calle 43 No. 130, Colonia Chuburná de Hidalgo, Mérida C.P. 97200, Yucatán, Mexico; LCRZ@cicy.mx

**Keywords:** nucleolus, intrinsically disordered, phase separation, fibrillarin, phosphoinositides

## Abstract

The process of phase separation allows for the establishment and formation of subcompartmentalized structures, thus enabling cells to perform simultaneous processes with precise organization and low energy requirements. Chemical modifications of proteins, RNA, and lipids alter the molecular environment facilitating enzymatic reactions at higher concentrations in particular regions of the cell. In this review, we discuss the nucleolus as an example of the establishment, dynamics, and maintenance of a membraneless organelle with a high level of organization.

## 1. Introduction

Cellular compartmentalization through lipid membranes permits specific cellular processes to take place at distinct regions inside the cell [1]. However, highly complex cellular systems require a rapid interchange of reaction components of biochemical processes with low energy consumption. A growing body of evidence shows that membraneless organelles display highly ordered structures and possess functional features similar to membrane-bound organelles [2,3,4]. Cellular membraneless organelles are involved in fundamental and dynamic processes, such as ribosome biogenesis, transcription, and RNA processing, and their behavior is receiving more attention due to their molecular principles [5]. Membraneless organelles display a liquid-like behavior, a characteristic of liquid–liquid phase separation (LLPS) [4,6,7]. Membraneless organelles are enriched in proteins, RNA molecules, and lipids [5,6]. These molecules, especially proteins, undergo regulation that can bear several modifications, which in turn modulates their propensity to interact with other components inside them. RNA constituents are important regulators of the formation and disassembly of membraneless bodies [7,8]. Maintenance of membraneless organelles (also called biocondensates) through the cell cycle remains elusive, and many questions arise about the nature of LLPS that takes place in a nonequilibrium system, e.g., a cell. The nucleolus provides a well-documented example of an organelle generated by phase separation [9]. Described as a factory of ribosomal RNA and ribonucleoparticles (RNPs), the nucleolus has to deal with a crowded microenvironment to accomplish these vital processes [10,11]. However, another large number of activities has been described for the nucleolus: stress response [12], cell cycle [13], genome integrity and stability [14,15,16], and RNP biogenesis [17]. All these events converge in a non-bounded membrane organelle, suggesting strict control of the molecular events driving each process. Moreover, how well-organized functional structures are formed to carry all nucleolar functions with a minimum energy requirement is still elusive.

Basic thermodynamics supports the principles that govern the formation and stability of biocondensates, but how epigenetic chromatin regulation, transcription, and other energy-dependent processes influence LLPS remains unknown. Nucleolar proteins and their unstructured regions, such as intrinsically disordered regions (IDR), are important drivers of LLPS. IDRs are amino acid stretches with non-conserved tridimensional structures, highly active inside biocondensates. Proteins with IDRs are present from prokaryotic to eukaryotic cells. Proteins with IDRs are generally composed of modules, i.e., conserved 3D structures with enzymatic activity and interspersed or flanked by IDRs. For instance, fibrillarin, which is a conserved enzyme in plants and animals, including mammals and humans, is composed of several structured domains and at N-terminus, it has glycine–arginine rich IDR that targets it to the nucleolus [18,19]. In this review, we focus first on the basic thermodynamic principles that drive LLPS in a nonequilibrium system, and then we will discuss the relevance of some key nucleolar IDR-containing proteins and their dysregulation related to diseases. We will also discuss the role of IDR-containing nucleolar proteins in processes demanding high energy consumption, such as cell division and rRNA transcription.

## 2. Basic Thermodynamics of LLPS Driving Nucleolus Assembly

The process of LLPS is an entropy-driven event and, therefore, described by the thermodynamic law of free energy state [20,21]. Entropy-driven events are defined as spontaneous processes taking place without consuming energy. As stated by the second law of thermodynamics, spontaneous reactions occurring inside the cell take place by lowering the free energy state in pressure and temperature constants. The formation of liquid-like droplets is a passive process that involves weak intra- and inter-molecular interactions between RNA, proteins, and lipids within a dynamic scaffold [2,3,22,23,24]. LLPS-assembled biocondensates typically display an almost spherical shape, propensity for fusion and/or fission, and a high degree of internal molecule dynamics [25,26]. Moreover, LLPS is required for processes related to rRNA transcription [11], mRNA metabolism [27], posttranslational modifications (PTM) of proteins, and signaling. For example, nucleolar structure formation by coalescence assembly involves two major driving forces: a passive thermodynamically-dependent process and an active energy-consuming process [28,29]. The two specific features of the droplet formation model by coalescence are temperature-dependence and reversibility. LLPS is a spontaneous mechanism that builds up the nucleolar region and possibly mediates the functional formation of a nucleolus by spinodal decomposition primarily in the free Gibbs energy. The process of spinodal decomposition involves a thermodynamically unstable solution that can transform within a miscibility gap to a mixture of two phases, since it occurs in a thermodynamically unstable state. The spinodal region of the phase diagram for each compound is where the free energy can be lowered by allowing the components to separate. Therefore, this results in an increased relative concentration of a component material (RNA, protein, or lipid) in a particular region of the cell. The concentration will continue to increase, thus, forming a particular structure (nucleoli, Cajal body, etc.). Once nucleation begins, a structure increases in size. To maintain the dynamics of the molecular characteristics of proteins, RNA and lipids must change by chemical modifications. Phosphorylation and methylation are some of the most common chemical alterations of these three types of molecules.

Very large regions of material will change their concentration slowly due to the amount of material that must move following passive processes, from high to low gradient concentration, isoelectric point, and electrostatic gradients. Very small regions composed of just a few molecules will shrink away due to the energy cost in entropy to maintain an interface between two dissimilar component materials [30,31,32]. It does not require nucleation events to form as the phases evolve continuously, thus, creating a separation of molecules without the aid of chemical energy. However, it is interesting to note that not only does LLPS mediate the formation of the nucleolus, but energy-consuming processes are also involved. Therefore, the enzymatic process is the second force that contributes to the formation of a functional nucleolus [17]. LLPS is dependent on the multivalent crosslinking interactions of molecules, such as RNA and proteins, that possess unstructured metastable regions or intrinsically disordered regions (IDR) [8,33]. These structural features give rise to typical properties of membraneless biocondensates, such as viscoelasticity and the fusion dependent on temperature [30,31,32]. Defining the energy required in the unmixing binary or ternary mixtures is not the only phase occurrence where energy-free growth occurs. Consider an example of two independent protein–lipid complexes, which undergo a particular order structure in thermal equilibrium. The phase transition forms a disordered state, where the two molecule species A and B are randomly distributed in the nucleolus forming an ordered arrangement depending on their concentration, thus creating a particular phase as described earlier by theoretical spinodal order, [34]. Phase separation of proteins that interact with nuclear lipids appear to form a pattern structure where each molecule occupies a defined position; therefore, the diffusion would follow a similar pattern as a spinodal decomposition.

**Figure 1:**
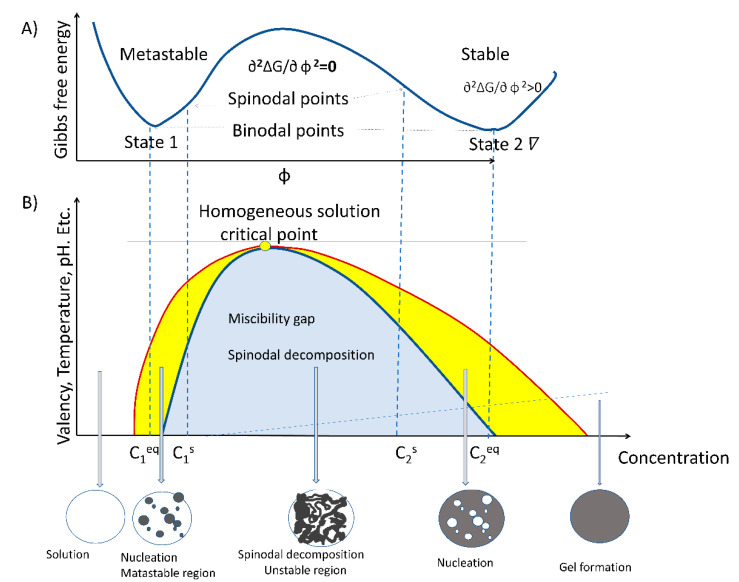
Schematic Gibbs free energy graph and the corresponding phase diagram. (**A**) Gibbs free energy graph shows the binodal and spinodal point where ∂^2^∆G/∂φ^2^ can be greater than zero or equal to zero, respectively; this illustrates the metastable regions before the spinodal points where instability takes place, followed by stable gel or precipitate formation. (**B**) The coexistence line (RED) separates the one-phase and two-phase regimes and is a function of environmental conditions, such as temperature and pH. The system does not undergo phase separation beyond the critical point marked as a blue dot. At low concentrations, the system is in the one-phase solution. Passing into the yellow zone, we have the binodal section where nucleation can take place; this is higher still in concentration between the spinodal points, where ∂^2^∆G/∂φ^2^ = 0 indicates the area region of instability in which the system must undergo demixing via spinodal decomposition.

However, nucleation also plays a role, under different concentrations for some of the compounds. In addition to the complexity of the mixture of materials, we have to consider the additional complexity of molecule modification by phosphorylation, acetylation, methylation, etc. [35]. All this alteration may allow a particular molecule to change its physicochemical characteristics, and since it would be at a different concentration, it may change position as it moves from a spinodal to a binodal or outside the range of phase separation. Thus, a molecule can transit from a particular structure to another without the addition of chemical energy. Figure 1 shows the miscibility gap where the initial free energy G would allow for a particular molecule of C_1_ concentration to phase separate from solution. The unstable zone where the spinodal decomposition lies is where the tangent of the curve from the Gibbs free energy changes so that the ∂^2^G/∂ Φ ^2^ = 0. Therefore, molecules can phase separate freely in this region and form particular structures without additional energy. In the cell nucleus, there is an added complexity of molecules, such as RNA, that may form nucleation events that can influence the range of concentrations for phase separation. Moreover, some of the molecules may have “fixed” positions due to interactions with specific DNA sequences. Nucleation requires additional energy, as stated in ∂^2^∆G/∂ φ ^2^ > 0. This may range from lncRNA to DNA binding proteins to set the particular locations for nucleation to take place. LncRNA may provide an additional dynamic situation. Previous studies have shown that RNA can alter the concentration required for phase separation for particular proteins [36,37]. Furthermore, the addition of RNA inhibitors, such as actinomycin D or DRB, result in nucleolar alteration and form different phase separations, forming the well-known nucleolar CAP [38,39]. Therefore, the range of concentrations in which a molecule can freely form a particular phase may be wider in the presence of RNA than if the compounds were taken in an in vitro scenario. These dynamics may prove relevant for nuclear organization, in particular, during the cell cycle as well as during temperature stress, where particular proteins, such as fibrillarin, that participate in the nucleolar process may redistribute to the cytoplasm [40]. Novel techniques that involve activation or bleaching of fluorescent labels are providing in vivo data required to define the thermodynamics of phase separation and complex formation in living cells under different conditions. Thus, there are some initial mathematical models for the diffusion of a particular volume fraction of a molecular protein population. Cahn and Hilliard derived a diffusion equation, which in its linearized form reads ∂φ_B_/∂ t = m[(∂^2^∆Gm/∂φ^2^_b_)∇·^2^φ_B_ − 2K∇·^4^Φ_B_] [41], where *K* is the energy gradient coefficient and *m* is the mobility coefficient. *D* is the diffusion coefficient *m*∂^2^ΔGm/∂·φ^2^_b_; therefore, the equation reduces to Fick’s diffusion equation. However, since ∂2ΔGm/∂·φ^2^_b_ is negative within the spinodal region, *D* must also be negative. Therefore, phase separation must occur by a diffusional flux against the concentration gradient in the spinodal region, thus behaving as an uphill diffusion. Again, diffraction of lattices or fluorescent analysis will provide more data in the future to improve nonlinear dynamics in living systems to adjust dynamics to a complex scenario [42].

## 3. The Membraneless Nucleolar Compartment

**Figure 2:**
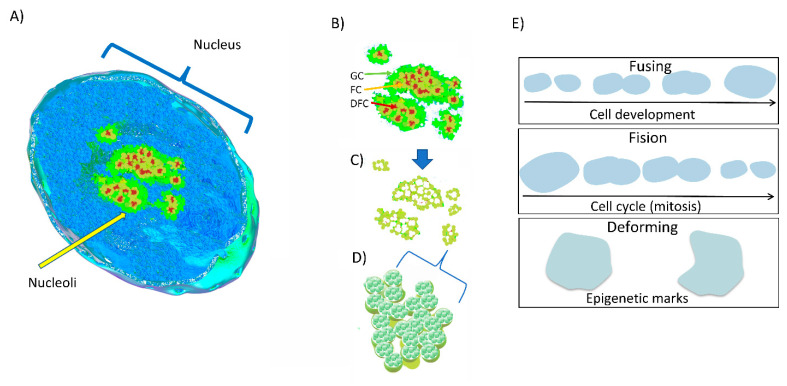
The multiphase structure of nucleolus. Our understanding of the behavior and composition of the nucleolus has been changed in recent years, as shown in (**A**) where key insights came from SIM and dSTORM wide field microscopy analysis to yield a more precise composition of the molecular crowded nucleolus. (**B**) shows key concepts regarding the LLPS behavior of the nucleolus. (**C**) Shows the ring-shaped structure organizing the components on (**B**). (**D**) Shows a close up of the elementary partition of each protein complex organizing the nucleolar hubs. (**E**) As biocondensates, the nucleoli can fuse to one another during cell development, can be broken into small hubs during mitosis, or change their form during specific epigenetic marks to coupling rRNA metabolism.

The nucleolus is a prototypical membraneless compartment inside eukaryotic cells and the location of several important processes during the cell cycle. The nucleolus in conjunction with other subnuclear structures, such as nuclear speckles, paraspeckles, PML bodies, lipid islets, or Cajal bodies, drives crucial, specific, and tightly regulated processes of RNA metabolism and processing. The nucleolus, as the core biocondensate drives the synthesis, processing, and assembling of rRNA into ribosomal proteins (r proteins) to produce functional ribosomes [10,43,44,45]. The nucleolus has a multilayer organization composed of three subcompartments: the fibrillar component (FC), the dense fibrillar component (DFC), and the granular component (GC) (see Figure 2). A recent study describes a fourth subcompartment around the GC: the nucleoli rim [46].

Nevertheless, the nucleolar structures have several mysteries to be resolved. The addition of DRB, a well-known RNA pol II inhibitor, results in a particular change in shape of the nucleoli, which suggests some particular RNA produced by these enzymes, is required for the structure of the nucleolus. However, it is very different from the addition of actinomycin D, as an RNA pol I inhibition, where the structure of the nucleolus tends to shape itself as a semisphere with two smaller spheres (known as CAPs) attached. Separation of these phases indicate that Actinomycin D may present a crucial component for the structure of the nucleolus [40]. In particular, to understand how the nucleolus forms during the cell cycle, nucleolus assembly occurs at the end of mitosis and proceeds until interphase. The nucleolus disassembly begins with the mitosis onset in each cell cycle. During embryogenesis in Drosophila melanogaster, the nucleolus formation depends on a seeding process, known as nucleation, in which the presence of rDNA and the active transcription of rRNA turn into highly saturated points to begin the nucleolar assemble. Nucleation is a highly stochastic process and the first step to building up the nucleolus. Moreover, nucleolar assembly is strictly dependent on rRNA transcription, as its inhibition blocks the coalescence membraneless body formation [47,48,49]. The presence of rDNA is not a prerequisite to assemble a highly saturated point as demonstrated in D. melanogaster in which the nucleolar protein fibrillarin and the RNA polymerase I 135kD subunit (Rpl135 RNA Pol I) factor still congregate at a homogeneous point into the nucleoplasm. Recently, it was demonstrated that Pol I activity is negatively regulated by an LLPS process [49]. The authors evaluated the dynamics of the major RNA polymerase I 194 kDa subunit (RPA194) and the chromatin upstream binding factor (UBF) by single-molecule tracking. In the presence of the transcription inhibitor CX-5461 drug, the dynamics of Pol I bounded to chromatin changed to a dissociated form in the same way as a RPA194 E593Q mutant, causing cranioskeletal malformation syndrome. This dissociation led to a new subcompartment with LLPS behavior, designed as the nucleolar cap where the Pol I molecules are more diffusive. The nucleolar structure is related to high rRNA transcription activity. Although the nucleolar structure has been elucidated at the superresolution level, the complete nucleolar organization is still sparse. This could rely on the formation of a structural platform by interacting factors, such as UBF and RNA Pol I machinery factors, that shape the classical three subcompartment distributions of the nucleolus. Using 3D structured illumination microscopy (3D-SIM) ring-shaped structures were visualized with UBF coupled to green fluorescent protein (GFP) and immune-stained by nanoantibodies. Moreover, the largest Pol I subunit RPA194 displayed a ring-shaped structure as visualized by Three-dimensional Structured Illuminated Microscopy (3D-SIM). The data suggest that single ring structures of UBF correspond to single transcription units looped in a way that excludes the intergenic spacers (IGS) between each rDNA unit but the possibility of two rDNA repeats actively transcribed is not discarded [47]. Current studies provide more data as to the composition of each part that phase separates during this process. Nevertheless, the molecular model of DNA pol I transcriptions still requires further tuning and new techniques to access the role of RNA as a structural component. Moreover, the dynamics of pre-ribosome structures from the GC to be completed elsewhere require further elucidation. Additional problems may rise with the generation of tailormade ribosomes and ribosomes that are altered in their structure by a different composition of methylation sites. This hack has been performed by some organisms, but it is also a relevant condition to study in cancer cells where highly methylated ribosomes surpass IRES leading to misread translation that results in some types of cancers [50].

## 4. Intrinsically Disordered Proteins Promote LLPS in the Nucleolus

Even though the different biocondensates are crowded, some of the key structural features are promoted by a certain set of proteins known as scaffolds, and the increasing body of proteins that binds them are known as clients. Scaffold and client interactions promote the organization and maintenance of structures, such as P-bodies [39] and PML nuclear bodies. The key features underlying the organization of these bodies reside in the stoichiometry of their scaffolds and the valency of clients, as demonstrated by Banani and coworkers [2] using a small set of three combinatory proteins known to phase separate in vivo and in vitro rRNA transcription, and the following modifications of rRNA involve several proteins and modifying complexes. In this section, we discuss the topics driving the current paradigm of LLPS in the nucleolus as the major biocondensates described: (a) ID proteins and ID regions; (b) motifs and physicochemical features present in IDR; (c) posttranslational modifications regulating the activity of IDR, and (d) key examples of nucleolar proteins regarding LLPS.

### 4.1. Intrinsically Disordered Proteins and Intrisnically Disordered Regions

**Figure 3:**
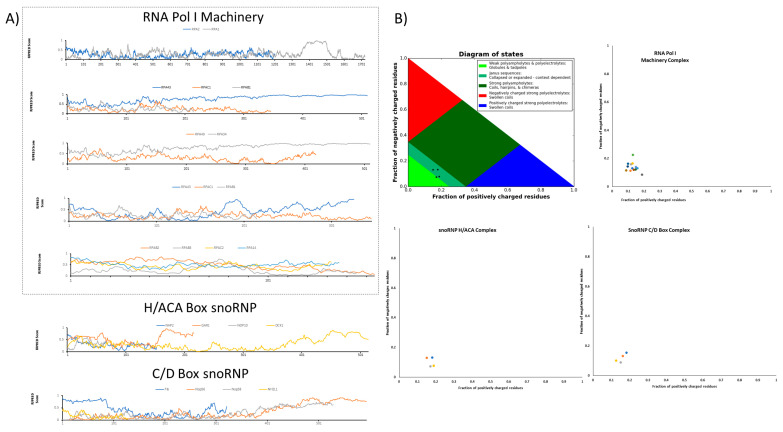
Intrinsically disordered regions in some key protein components during rRNA metabolism drive the LLPS behavior of the nucleolus. (**A**) The intrinsically disordered tendency of the RNA Pol I machinery and the well-characterized snoRNP complexes driving pseudo-uridylation and methylation, snoRNP H/ACA, and snoRNP C/D Box, respectively. As calculated by IUPred algorithm (www.iupred2a.elte.hu, accessed on 11 March 2021), a tendency to disorder is predicted when values per residue exceed the 0.5 IUPred scores. Intrinsically disordered regions vary between complexes and between N- and C-terminal domains, for example, the major RPA1 factor of RNA Pol I exhibit a tendency to disorder in its C-terminal domain and the GAR1 protein founded in the H/ACA complexes. In contrast, fibrillarin from the C/D Box complex displays a well-documented disordered region in its N-terminal domain. (**B**) Diagram phases computed with the CIDER (Classification of Intrinsically Disordered Ensemble Regions; www.pappulab.wustl.edu/CIDER, accessed on 11 March 2021) algorithm showing the expected conformation that IDP could adopt ranging from globules and tadpoles, collapsed or expanded, coils and hairpins, and swollen coils. Although the disordering tendency shows a set of proteins containing IDR, each corresponding diagram phase indicates a small tendency to phase separate. As shown in the phase diagrams, the majority of the proteins involved in the complexes fall in the phase two plot region, as a collapsed and extended region. This corresponds to the idea that LPPS is coupled to other energy-consuming processes that in the last step promotes a multicomponent phase-separated nucleolus. Protein names were omitted in the plots to prevent overlapping.

Nucleolar proteins often contain enzymatically active regions flanked by IDRs (see Figure 3). These intrinsically disordered regions in their amino acid sequences are coupled to a modular region with enzymatic activity, RNA-binding regions, or nucleolar localizing signals. Throughout this section, we want to clarify that enzymes are generally known by their 3D conserved structure, but recently our understanding of structure and function are changing due to new concepts and paradigms in molecular biology, such as LLPS. Now, proteins are currently categorized in two main groups: (a) well-structured proteins and (b) proteins with non-conserved 3D, subdivided into fully disordered proteins and proteins with IDR. Nucleolus-residing proteins are well characterized by their contribution to sorting, modifying, and assembling rRNA and ribosome production. Often, nucleolar proteins contain stretches of IDRs in their sequences, highlighting that IDRs are important to drive the synthesis and sorting of rRNA transcripts. We will discuss in the following paragraphs important insights to establish and maintain biocondensates.

### 4.2. Physiochemical Features and Sequences Signatures in IDR-Containing Proteins

The tunable physicochemical properties of key nucleolar proteins involved in several aspects of cell cycle and cell development had raised questions about how PTMs could explain how biocondensates are regulated in an active energy-consuming process. Several proteins related to rRNA transcription, processing, and nucleolus assembling possess low complexity domains often called intrinsically disordered domains, characterized by aromatic and charged amino acids. Here, we discuss some of the most abundant proteins inside the nucleolus known for their roles in rRNA metabolism. A well-documented signature is the RGG/RG repeat, characterized by the fact that this region is modified by methyltransferases, adding a methyl group to the terminal guanidine nitrogen atom of the arginine. Fibrillarin and nucleolin are highly expressed proteins in the nucleolus and contain stretches of RGG/RG repeats in their sequences [51,52,53].

### 4.3. Posttranslational Modifications Regulating IDR-Containing Proteins

However, how these RGG/RG signatures impact the fluidity and maintenance of subnucleolar organization is still obscure. Arginine methylations inhibit LLPS in vitro, but in vivo arginine methylation promotes LLPS by two mechanisms: (1) modifying protein–RNA interactions or (2) promoting partner interactions between proteins. Thus, in vivo, arginine methylations seem to promote LLPS rather than inhibit it. Phosphorylation of serine or tyrosine residues has been getting more attention for its relevance in LLPS [24,54]. Contrary to arginine methylation, phosphorylation and dephosphorylation of threonine and tyrosine residues can occur quickly and change the properties of biocondensates in a tunable manner. Further information about PMT as key regulators of LLPS can be found in the excellent review by [54].

### 4.4. Key Nucleolar Proteins Containing IDR

There is evidence regarding the influence of key nucleolar proteins residing in the nucleolus whose dysregulation could impact the LLPS maintenance of nucleolar structure; these proteins are fibrillarin, nucleolin, GAR1, and nucleophosmin, among others. Nucleolin is a highly conserved protein found in the nucleolus GC, composed of three domains: an N-terminal domain integrated by HMG-like domain, a central domain with two different stretches of RNA binding domains, and a C-terminal domain rich in arginine and glycine repeats similar to that found in fibrillarin [55,56,57]. Two main proteins residing at the DFC, fibrillarin, and GAR1 are the catalytic centers of two different complexes that guide the specific site methylation of ribosomal RNA (rRNA) nucleotide residues and the pseudo-uridylation of uridine residues in the rRNA, respectively [58]. These two proteins contain IDR at the N- and C- terminal regions, respectively [46,53]. The N-terminal IDR of fibrillarin is a glycine–arginine rich region (GAR-domain) containing several repetitions of [RG] boxes in the whole domain [59]. The fibrillarin GAR-domain functions as a signal that directs the protein to the nucleolus to produce ribosomes, and this region is also involved in the recognition of lipids, some specific RNA partners, and other proteins. Recently the GAR-domain of *Arabidopsis thaliana* fibrillarin was described as a modular domain with ribonuclease activity, adding another layer of complexity to this domain and the whole protein itself [60]. The GAR1 protein is the central catalytic unit of the H/ACA ribonucleoparticle complexes, mainly involved in the modification of specific residues on the rRNA, but it is not clear if the C- terminal IDR is playing an important role in the interaction with other partners and localization in the nucleolus [58]. Nucleophosmin (NPM1 also B23) is a highly conserved nucleolar phosphoprotein related to the last stages of ribosome biogenesis and located at the GC [61]. As other nucleolar proteins, NPM1 is a modular protein composed of several domains: an oligomerization domain (OD) and two IDRs located between the OD and the C-terminal domain, which possess DNA and RNA binding activity, binding G-quadruplex DNA sequences [61,62]. NPM1 functions are often related to the last steps in ribosome processing and assembly with r-proteins, but also several other functions of NPM1 were described: regulation of centrosome duplication, genome stability, stress response, apoptosis, cancer, and rRNA gene remodeling. Although NPM1 is important for rRNA assembly with r proteins, its function is indispensable for ribosome biogenesis. NPM1 depletion disrupts the nucleolar structure. Nucleolar localization of NMP1 depends on the formation of a pentamer oligomer, and the disruption of this by a single amino acid phosphorylation on the serine 48 located in the OD changes its localization to the nucleoplasm. Oligomerization relies on OD and on the adjacent acidic IDR [63]. Further, lipids are important in the LLPS system [8,40,64]. It remains unclear how lipids could be involved in the formation, nucleation, or maintenance of biocondensate bodies. Recently, the nuclear nanoscale localization of PI(4,5)P2, PI(3,4)P2, and PI(4)P was found by dSTORM. A subset of PI(4,5)P2 and PI(3,4)PI was detected near the RNA Pol II machinery, specifically on nuclear speckles, a biocondensate known to concentrate mRNA and proteins related to RNA modifying routes. Moreover, another population of PI(4,5)P2 was detected in the nucleolar DFC, surrounded by fibrillarin, displaying a ring-shaped structure [65]. Lipid islets have been related to the creation of nucleation points that concentrate key signal proteins in response to several types of stress [64]. RNPs are well conserved complexes in eukaryotic cells, mainly concentrated where modifications and the last processing events of the ribosome biogenesis take place. Classic examples correspond to the C/D and H/ACA Box RNPs, enrolled with methylation and pseudo-uridylation of specific residues on the recently transcribed rRNAs (see Figure 3) [66,67,68,69]. The link between RNPs and their content of intrinsic disorder on their protein components has been demonstrated (Figure 3), for example, the GAR1 and fibrillarin proteins for H/ACA and C/D box RNPs [52,70]. Moreover, it has been demonstrated that the presence of IDR in the RNP complex drives LLPS events inside the cell [71,72]. Studying RNP biogenesis as well as its functional activity inside the cells could shed some light on the principles governing LLPS, because RNPs can form specific and focal volume of nucleation points that resemble the major LLPS biocondensates found in cells. A clear example is the nature of the RNP complex in which the uL3 expression status could also influence rRNA biogenesis in certain types of cancer [73].

## 5. LLPS and Disease

Although in past decades the link between how membraneless organelles and biocondensates are related to diseases was obscure, some light has been shed on the mechanisms in which the transition and, more specifically, the aberrant transitions from a liquid-like to solid-like state promote pathologies such as cancer, viral infections, and other complex diseases, such as neurodegenerative pathologies as Alzheimer’s disease, Amyotrophic lateral sclerosis, Parkinson’s disease, and Huntington’s disease [74,75,76]. In addition, with the advent of more research and compression of the principles governing LLPS, new avenues in the diagnosis, treatment, and early detection of these pathologies could be improved. The mechanism by which diseases and LLPS are linked remains elusive; even so, some clues suggest the hypothesis that changes in the environmental micro conditions in the cell, dysregulation of some specific factors, enzymes, or the molecule thermodynamics itself could lead to aberrant biocondensate functionality. For example, fused in sarcoma (FUS) protein, a well-documented protein related to mRNA splicing and transcription, has been demonstrated to phase separate in vitro and in vivo; indeed, it possesses prion-like low complexity domains in its sequence. Recently, it was shown that the transition of dynamically active liquid separation of FUS protein to a more solid and rigid state is determinant for amyloid lateral sclerosis (ALS), and mutations in patients with ALS revealed that intrinsically disordered domains could explain this transition [77]. Although this is the first evidence on how prion-like intrinsically disordered domains and LLPS are involved in disease, some other questions are arising because FUS proteins form functionally active bio-condensates as P granules and also colocalize in nucleoli, and the tightly regulated events necessary to prevent to become a more rigid and solid state (known as hydrogels) are not fully understood [77,78]. Dysregulation of RNA-binding proteins is often related to aging diseases, such as frontotemporal dementia and ALS, which possess fibrillar-like markers in affected cells. The clues came from key proteins, as the heterogeneous nuclear ribonucleoprotein (hnRNP) A1. hnRNPA1 can undergo phase separation by its intrinsically disordered domain but is not sufficient to drive LLPS in an RNA body context. Indeed, hnRNPA1, as do other proteins related to RNA metabolism, possesses multivalent RNA-recognition and binding motifs with low complexity that are key in the maintenance and functionality of RNA-protein biocondensate bodies [79]. A T cell-restricted intracellular antigen-1 (TIA1) protein was described as a molecular marker of degenerative disease, and the mutations related in its low complexity domain are signatures of disease. It is critical to note that these mutations affect the solubility and dynamics of TIA1, promoting LLPS in a liquid-like body [80]. The changes in the thermodynamically protein behavior in some diseases is linked to a lack of regulation of LLPS. Widespread cancer is another pathology recently described by a LLPS aberrant transition in its molecular mechanism [81,82]. The tumor suppressor SPOP (speckle-type POZ protein) was demonstrated to localize into liquid nucleolar bodies, and mutations in cancer-specific amino acids W131G and F33V disrupt this localization.

### Nucleolar Components and Cancer

Since their discovery more than 200 years ago, nucleoli have been associated with health and development stability in the cells. However, the number and shape of nucleoli inside the cells are often related to a high protein demand synthesis. In fact, the aberrant shape and number in different types of cancers have been associated and function as morphological markers of disease. Although, ribosome biogenesis is the central role of nucleoli, other secondary tasks are dependent on a healthy nucleolus, such as: cell cycle progression, stress response, genome stability, and maintenance, among other functions. Cancer conditions and related deregulations are often associated with the main and prominent function of rRNA transcription, rRNA modification, and ribosome production driven by nucleoli [79,83]. Dysfunctional RNA Pol I activity associated with cancers has been detailed and reported previously [49,84,85,86] but, in fact, is not only the canonical function of nucleoli driving the rRNA synthesis, the principal event promoting cancer. In fact, the secondary or noncanonical functions of nucleoli have been linked to cancer, describing noncanonical functions relevant to the dysregulation of functions found in several types of cancer [80,82,83]: (1) dysregulation of cell cycle and maintenance of genome stability; (2) genetic alterations in ribosomal protein production; (3) dysregulation of nucleolar stress pathways; and (4) dysregulation of the production and function of ribonucleoparticles complexes. Here, we present a brief table (Table 1) showing some protein nucleolar components whose functions have been related to several types of cancers [87].

## 6. Conclusions

**Figure 4:**
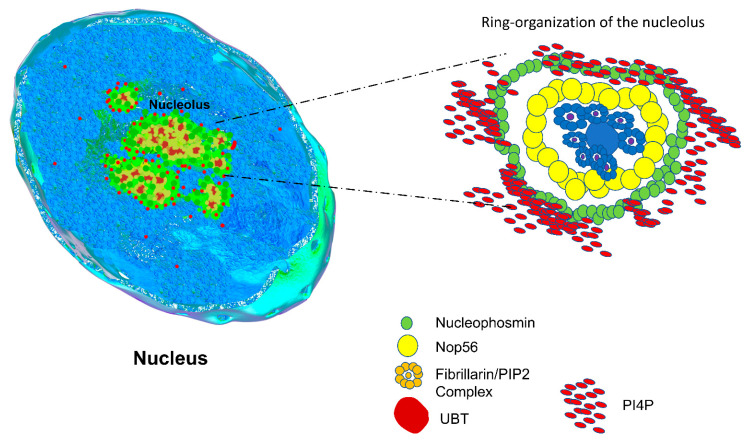
The rim organization of nucleolus. A more current view of the nucleolus as a biocondensate body includes a small-scale organization of specific molecules, as described in other studies for fibrillarin and PIP2 forming small nucleation foci inside the DFC, and also with other phosphoinositides (not shown in the figure). A ring structure appears as a scaffold to confer the properties of a highly dynamic multi-organized nucleolar body.

Cells rely on a multitude of compartments to conserve their fitness, development, and cell cycle. A particular scenario of compartmentalization comes with the growing evidence that biocondensates are regulators of key aspects of the biochemical and physiological status of the cell. How the interplay between IDR-containing enzymes, lipids, and RNA populations can regulate their formation and maintenance is still a relevant topic in cell biology, biophysics, and thermodynamics. An IDR possesses a high valency and propensity to bind other molecules. Its structure and binding capacities is restricted by its amino acid composition and its intrinsic thermodynamic properties but also modulated by the microenvironment in a defined volume of the cell. The relationship between IDR entropy and enthalpy properties related to the formation of a nucleolus as a biocondensate is still obscure, and more attention is needed in order to understand how IDR can regulate the nucleolar structure formation. In the present review, we summarized with a thermodynamic scope: (1) the formation of the nucleolus, the major biocondensate structure present in living cells; (2) the relationship between nucleolar enzymes possessing IDR in their sequences and diseases, and (3) the current view of ring structures observed with new visualization strategies and tools. Understanding the formation of structures, such as the nucleolus from a thermodynamic view, will increase our knowledge about how diseases are dysregulating LLPS properties, and how cells can couple high energy demand processes with passive phase separations. Finally, the paradigm of compartmentalization with a membrane enclosure and functionality is changing, because LLPS has emerged as a process driving not only the sequestration of molecules in a defined volume but also functional and active regions inside the cell (Figure 4).

## Figures and Tables

**Table 1:** Protein nucleolar component related to clinical disease. Nucleolar proteins are often dysregulated in several types of cancers and important clinical diseases. A brief list of examples is described in the table with the functional activity inside the cell and the dysregulated activity presented in the disease.

Nucleolar Protein	Link to Disease	Cellular Function	References
Nucleolin	Dysregulation of RNA binding activity	rRNA synthesis	[88,89,90]
Nucleophosmin	Dysregulation of nucleolar protein activity	rRNA synthesis	[63,91]
Fibrillarin	Dysregulation of methyltransferase activity	rRNA synthesis/Ribonucleoparticle complexes	[92,93]
GAR1	Dysregulation of pseudo-uridylation activity	rRNA synthesis/Ribonucleoparticle complexes	[70,94]
PPAN	Dysregulation of signaling pathway	Cell cycle and development	[95,96]
p14ARF	Dysregulation of signaling pathway	Cell cycle and development	[97,98]
uL3	Dysregulation of signaling pathway	Ribosomal protein	[73,87]
hnRNPH1	Dysregulation of signaling pathway	Nucleolar stress pathway	[99,100]
KHSRP	Dysregulation of signaling pathway	Nucleolar stress pathway	[101,102]
P53	Dysregulation of signaling pathway	Nucleolar stress pathway	[103,104,105]

## Data Availability

Not applicable.
